# Wavelet-promoted sparsity for non-invasive reconstruction of electrical activity of the heart

**DOI:** 10.1007/s11517-018-1831-2

**Published:** 2018-05-12

**Authors:** Matthijs Cluitmans, Joël Karel, Pietro Bonizzi, Paul Volders, Ronald Westra, Ralf Peeters

**Affiliations:** 1 Department of Data Science and Knowledge Engineering, Maastricht University, Maastricht, The Netherlands; 2 CARIM School for Cardiovascular Diseases, Maastricht University, Maastricht, The Netherlands

**Keywords:** Electrocardiographic imaging, Regularization, Cardiology

## Abstract

We investigated a novel sparsity-based regularization method in the wavelet domain of the inverse problem of electrocardiography that aims at preserving the spatiotemporal characteristics of heart-surface potentials. In three normal, anesthetized dogs, electrodes were implanted around the epicardium and body-surface electrodes were attached to the torso. Potential recordings were obtained simultaneously on the body surface and on the epicardium. A CT scan was used to digitize a homogeneous geometry which consisted of the body-surface electrodes and the epicardial surface. A novel multitask elastic-net-based method was introduced to regularize the ill-posed inverse problem. The method simultaneously pursues a sparse wavelet representation in time-frequency and exploits correlations in space. Performance was assessed in terms of quality of reconstructed epicardial potentials, estimated activation and recovery time, and estimated locations of pacing, and compared with performance of Tikhonov zeroth-order regularization. Results in the wavelet domain obtained higher sparsity than those in the time domain. Epicardial potentials were non-invasively reconstructed with higher accuracy than with Tikhonov zeroth-order regularization (*p* < 0.05), and recovery times were improved (*p* < 0.05). No significant improvement was found in terms of activation times and localization of origin of pacing. Next to improved estimation of recovery isochrones, which is important when assessing substrate for cardiac arrhythmias, this novel technique opens potentially powerful opportunities for clinical application, by allowing to choose wavelet bases that are optimized for specific clinical questions.

**Electronic supplementary material:**

The online version of this article (10.1007/s11517-018-1831-2) contains supplementary material, which is available to authorized users.

**Graphical Abstract:**
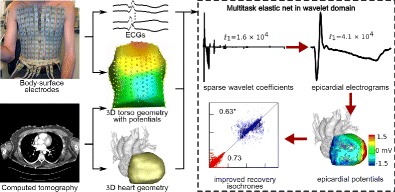
The inverse problem of electrocardiography is to reconstruct heart-surface potentials from recorded bodysurface electrocardiograms (ECGs) and a torso-heart geometry. However, it is ill-posed and solving it requires additional constraints for regularization. We introduce a regularization method that simultaneously pursues a sparse wavelet representation in time-frequency and exploits correlations in space. Our approach reconstructs epicardial (heart-surface) potentials with higher accuracy than common methods. It also improves the reconstruction of recovery isochrones, which is important when assessing substrate for cardiac arrhythmias. This novel technique opens potentially powerful opportunities for clinical application, by allowing to choose wavelet bases that are optimized for specific clinical questions.

## Introduction

Heart rhythm disorders are among the leading causes of deaths worldwide. The 12-lead electrocardiogram (ECG) is a well-established, patient-friendly, quick, reproducible, and cheap tool to determine normal cardiac activation and recovery, to diagnose cardiac arrhythmias, altered activation, ischemia, infarction, primary electrical abnormalities of the heart, structural disease, and other conditions. It reflects the attenuated and dispersed propagation of electrical activity of the heart on the body surface. However, it lacks the capacity to directly assess spatial electrical activity at the level of the heart muscle at high spatial resolution.

**Fig. 1:**
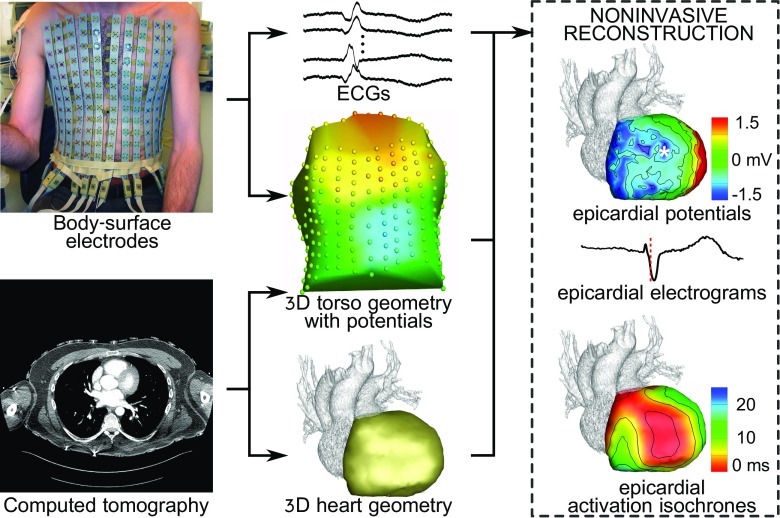
Electrocardiographic imaging (ECGI) non-invasively reconstructs electrograms and activation and recovery isochrones on the epicardium (outer heart surface). Body-surface ECGs are combined with a torso-heart geometry obtained with CT. By carefully reversing a model of the physical laws of electromagnetism, epicardial potentials can be reconstructed. From these, epicardial electrograms and isochrones are deducted

Electrocardiographic imaging (ECGI) non-1invasively reconstructs potentials, electrograms, and activation/recovery isochrones directly at the heart surface from body-surface potential measurements and a patient-specific torso-heart geometry [5, 7, 33, 34] (see Fig. 1). This is achieved by solving what is known as “the inverse problem of electrocardiography.” In the last decades, much progress has been made in ECGI and clinical applications are published with increasing frequency [7].

However, the accuracy of the reconstructed electrical heart activity is still suboptimal. This is partly due to the non-uniqueness and ill-posedness [13] of the inverse problem: the solution is not unique and small variations (noise and measurement errors) in the input data can lead to large variations in the reconstructions [7]. To cope with this problem, *regularization* is applied, i.e., additional knowledge is incorporated, in the form of constraints on the possible solutions, in order to arrive at more realistic results [22, 29]. For a review of common regularization methods in the field of ECGI, see [25, 30].

One way to overcome the influence of ill-posedness is to work with the data in a different domain. For example, in a previous study, we have shown that accuracy is improved when heart-surface potentials are determined as a function of physiologically realistic potential patterns obtained from numerical models that function as “building blocks” [6]. A drawback of that method and other commonly used methods [25, 30] is that they apply regularization only on the spatial distribution of potentials (at a certain time point) or only on the temporal behavior of potentials (at a certain spatial location). In this study, we propose to use a different sparse representation, in terms of wavelets. By using a wavelet basis, only few wavelet expansion coefficients are necessary to describe electrograms at the heart surface. This approach allows to achieve regularization over space and time simultaneously. Wavelets have been used in the inverse problem of tissue imaging, such as magnetic resonance imaging (MRI) [3], but not yet in the inverse problem of electrocardiography.

The purpose of this work was to investigate whether such a wavelet-promoted spatiotemporal regularization could improve accuracy of reconstruction of the electrical activity of the heart. We have a special interest in reconstruction of recovery times, which are currently more difficult to obtain with ECGI than activation times [5]. This is relevant because abnormalities in recovery form an important substrate for arrhythmias and are difficult to assess with current noninvasive tools. We evaluated our novel method in canine experiments for which simultaneous body-surface and invasive heart-surface recordings were obtained.

## Methods

**Fig. 2:**
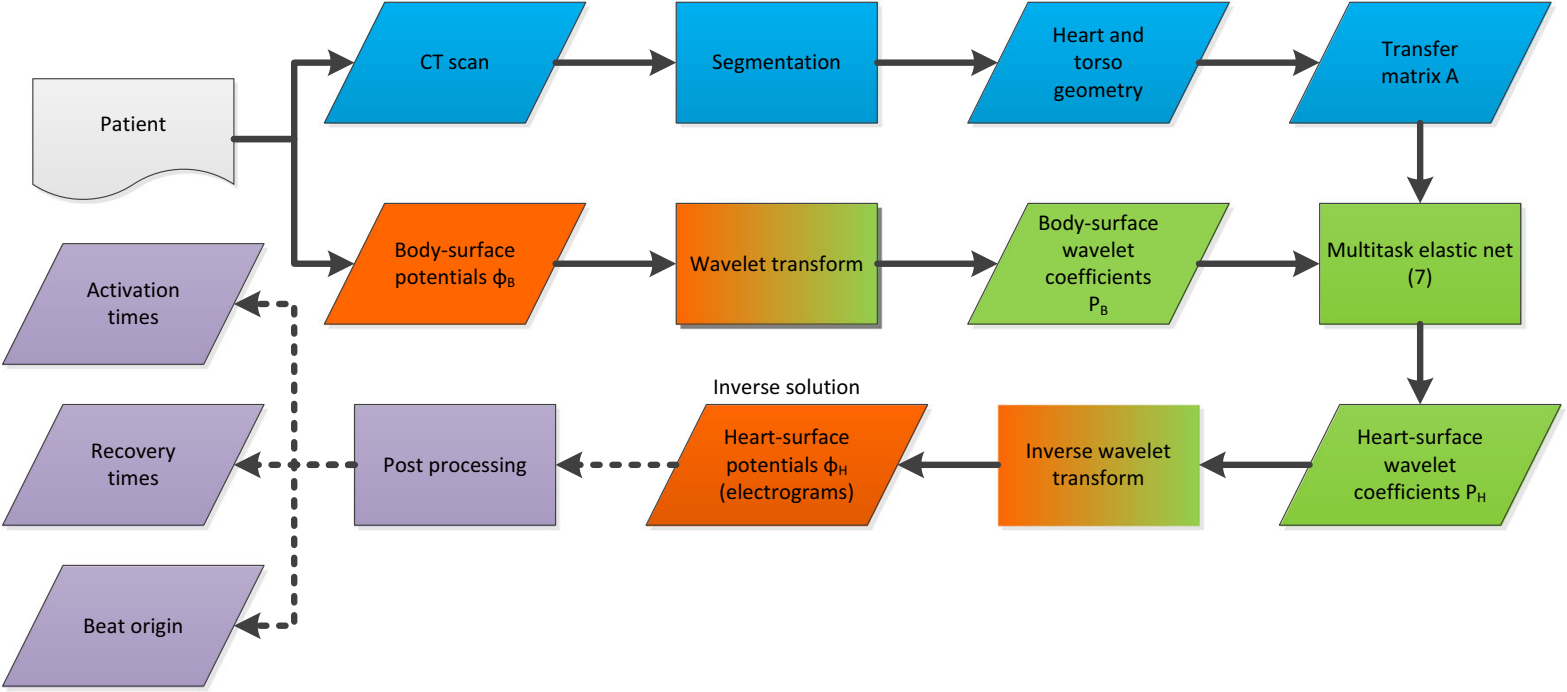
Schematic illustration of the inverse approach employed in this paper

The approach for finding the ECGI inverse solution as presented in this paper is based on working in the wavelet domain, obtaining a sparse solution with multitask elastic net and transforming back to the time domain. Figure 2 illustrates this approach and this is further discussed in detail in the following subsections.

### The inverse problem of electrocardiography

The potential-based formulation of the forward and inverse problems of electrocardiography is based on the assumption that there is a direct and instantaneous linear relationship between potentials at a closed surface surrounding the heart and the potentials at the body surface [30]. The closed surface surrounding the heart is usually taken to be the epicardium, i.e., the outer heart surface. The forward model describes the way that body-surface potentials are induced by heart-surface potentials. It is commonly defined as:

1 $$ {\Phi}_{B} = A {\Phi}_{H} + N  $$

where Φ_*B*_ is an *r* × *m* matrix of potentials at *r* body-surface nodes at *m* moments in time, Φ_*H*_ a *q* × *m* matrix of potentials at *q* heart-surface nodes at the same *m* time instants, and *N* is additive independent noise with equal variance. The *r* × *q* transfer matrix *A* depends on the geometry (the node locations, both on the torso and on the heart) and the medium (the conductivity properties of the body tissues). The model neglects the influences of tissue capacitance and any sources of electrical potentials other than the heart, as well as the effects of relative motion of the nodes (e.g., due to a contracting beating heart and breathing). It also assumes that the *q* heart-surface nodes are sufficiently densely generated to well represent all electrical potential on the heart. The matrix *A* is typically estimated based on a computed tomography (CT) scan of the patient’s torso with body-surface electrodes attached.

The objective of the inverse problem of electrocardiography is to find Φ_*H*_ under the linearity assumption in (1) with assumed Gaussian white noise *N*, so as to best explain a recorded instance of Φ_*B*_, and given the estimated transfer matrix *A*:

2 $$ \underset{{\Phi}_{H}}{\min} \left\| A{\Phi}_{H}-{\Phi}_{B} \right\|_{F}^{2}  $$

Here, the notation ∥⋅∥_*F*_ is used to indicate the entrywise 2-norm for matrices, i.e., the Frobenius norm. The choice of this norm is motivated by the time-invariance of the forward linear model, the identical technical specifications of the electrodes, and the assumed independence and (Gaussian) whiteness of the measurement errors.

Problem (2) is non-unique, as the number of heart-surface nodes *q* is generally taken to be much larger than the number of electrodes *r*, making matrix *A* not full rank. Matrix *A* is also ill posed, as the forward model is only approximate, and *A* can only be estimated with limited accuracy, due to necessary simplifications with respect to torso inhomogeneities, movement of heart and torso, or changes in conductivity.

Therefore, to obtain a stable solution to the inverse problem, additional constraints on the feasible solutions are needed, either implicit or explicit. For example, the well-known Tikhonov regularization method [40] does this implicitly. For an in vivo evaluation of Tikhonov-based reconstruction of epicardial potentials, see our previous study [5].

### Multitask elastic-net-based reconstruction of epicardial potentials with wavelet-domain regularization

Another approach to regularization is to reduce parameter redundancy by representing Φ_*H*_ sparsely. One may do this explicitly, by preselecting a limited number of “building blocks” to reconstruct Φ_*H*_, as in [6]. An implicit, more flexible way to obtain sparsity is by minimizing the least-squares error criterion under an *ℓ*_1_-norm constraint. The *ℓ*_1_-norm is widely used to promote sparsity [2], for example in the *total variation method* [27], the *lasso* method [39], or the more flexible *elastic-net* approach [16, 44], which offers a combination between lasso and Tikhonov regularization. Extending elastic-net to *multitask* elastic net additionally allows for exploiting structure between the spatial patterns at each time instance. In order to enforce a sparse representation, we will apply multitask elastic net in the wavelet domain.

#### Elastic-net-based reconstruction

The straightforward approach for solving the inverse problem with elastic net is to work on each of the *m* columns of the potentials matrix Φ_*H*_ independently, yielding *m* decoupled elastic net problems (each time instant processed individually):

3 $$\begin{array}{@{}rcl@{}} &&\underset{{\Phi}_{H}^{(k)}}{\min} \left\{ \left\| A{\Phi}_{H}^{(k)}-{\Phi}_{B}^{(k)} \right\|_{F}^{2}\right.\\ &&\left.+\lambda \left[\!(1\,-\,\alpha)\frac{1}{2}\left\| {\Phi}_{H}^{(k)} \right\|_{F}^{2} \!\,+\, \alpha\left\| {\Phi}_{H}^{(k)} \right\|_{1}\right]\right\}, \quad \!\!k\,=\,1,\ldots,m, \end{array} $$

where the superscipt ^(*k*)^ denotes the *k*-th column of a matrix, corresponding to the *k*-th time point, and ∥⋅∥_*p*_ the vector *p*-norm. In this case, the problem is decoupled over time, and sparsity is sought over space, per time instant. The constant *α* is the elastic-net mixing parameter, balancing between a lasso approach (*α* = 1) and zeroth-order Tikhonov regularization (ridge regression) (*α* = 0). The factor $\frac {1}{2}$ appears in convention with [10, 16] and the software glmnet [31]. Although the factor 1/2 before the Tikhonov term in the elastic net makes the resulting regularization not a convex combination of the two terms, this does not affect convexity of the problem in (6). Indeed, the L1 and L2 norms are convex functions, and any non-negative combination of those terms still defines a convex problem.

#### Multitask elastic-net-based reconstruction in the wavelet domain

Our assumption is that epicardial potentials Φ_*H*_ can be considered sparse due to the properties of propagating cardiac wavefronts. Indeed, only a specific part of the cardiac tissue is activated at a specific moment in time (sparsity over space at each time instant) and cardiac cells are activated according to propagation waves (sparsity over time at each location), causing the local electrograms to be sparse in both time and space. This justifies to pursue sparsity of Φ_*H*_ over time and space simultaneously. Furthermore, the frequencies that constitute these electrograms come from a limited frequency band, which is well represented in the wavelet domain. Therefore, we propose a method which combines an orthogonal wavelet transform as is commonly used in ECG signal processing [36] with multitask elastic net.

A discrete wavelet transform is a cascade of *N* filter banks, governed by a pair of wavelet filters, yielding *N* wavelet scales. Discrete-time wavelet transforms can also be represented by a convolution matrix^1^*W* [38]. This matrix *W* will be orthogonal if the wavelet transform is orthogonal, for which the underlying wavelet must be orthogonal, the transformation must be critically sampled and the borders must be handled in a way preserving energy; in this case, by periodic extension. By multiplying *W* with Φ_*H*_, one can obtain the matrix *P*_*H*_:

4 $$ P_{H} = {\Phi}_{H} W  $$

The matrix *P*_*H*_ collects in each row a set of scaling and wavelet coefficients of all the electrograms in the rows of Φ_*H*_, from coarse to fine. With the exception of the Haar wavelet transform, an orthogonal wavelet transform cannot be linear phase [9, thm 8.1.4].

Since multiplication by an orthogonal matrix does not change the 2-norm, and it is natural to require the matrix *P*_*H*_ to be sparse, the set of *m* decoupled elastic net problems in (3) can be rewritten as:

5 $$\begin{array}{@{}rcl@{}} &&\underset{P_{H}^{(k)}}{\min} \left\{ \left\| AP_{H}^{(k)}-P_{B}^{(k)} \right\|_{F}^{2} \right.\\ &&\left. +\lambda \!\left[(1\,-\,\alpha)\frac{1}{2}\| P_{H}^{(k)} \|_{F}^{2} + \alpha \| P_{H}^{(k)} \|_{1}\right] \right\}, ~~ k\,=\,1,\ldots,m, \end{array} $$

where *P*_*B*_ = Φ_*B*_*W* denotes the matrix of body-surface wavelet coefficients using the same orthogonal wavelet transform. When applying the wavelet transform, we choose for a redundant representation of the discrete wavelet transform, i.e., the undecimated wavelet transform, which is practically implemented here in the form of the stationary wavelet transform [37]. The advantage of having redundant information is that smoothness is better retained after regularizing. In this scenario, the number of columns of *P*_*H*_ is equal to the number of columns of Φ_*H*_ times the number of decomposition levels *N*. In case that the stationary wavelet transform is used, the matrix *W* is no longer orthogonal. However, if the used wavelet filter is orthogonal, the energy is weighted by a factor 2^*j*^ with increasing *j* as the scale becomes coarser [19, 20].

Concerning the choice of a suitable wavelet, electrograms can be sparsely represented with the orthogonal Daubechies-2 wavelet (2 vanishing moments, filter length 4) [19]. If desired, one may also design a more dedicated orthogonal wavelet by using the approach of [17, 20]. The Online Supplement (Online Figure 3) illustrates this.

In (5), the wavelet transform is applied over each row of Φ_*H*_ (over time only), to promote sparsity over time. Each $P_{H}^{(k)}$ thus denotes a vector of wavelet coefficients of index *k*, collected over all nodes at a specific time instant. This means that by solving for each $P_{H}^{(k)}$ independently, the problem gets decoupled over the wavelet coefficients (and thus time), and the natural relationship between the wavelet coefficients is ignored. For this reason, it is preferable to solve the *k* regression problems jointly, imposing some type of group structure on the coefficients (to take into account that groups of nodes have a similar time-frequency content). This can be achieved by combining the *m* elastic net problems into a single multitask elastic net problem [21, 26]. Joint sparse estimation of the coefficients can then be achieved by including a penalty term consisting of the *ℓ*_1_-norm of the root energies (*ℓ*_2_-norm) per wavelet coefficient over all time instants:

6 $$\begin{array}{@{}rcl@{}} &&\underset{P_{H}}{\min} \left\{\left\| AP_{H}-P_{B} \right\|_{F}^{2}\right.\\ &&\left.+ \lambda \left[ (1-\alpha) \frac{1}{2}|| P_{H} ||_{F}^{2} + \alpha \sum\limits_{k = 1}^{m} || P_{H}^{(k)} ||_{2} \right]\right\}. \end{array} $$

This promotes sparsity over all scales and wavelet coefficients (time-frequency) simultaneously, while exploiting the spatial group structure [16, 26]. The last penalty term of (6) is a mixed *ℓ*_2_/*ℓ*_1_-norm, i.e., the *ℓ*_1_-norm of the vector of columnwise *ℓ*_2_-norms of *P*_*H*_. The multitask lasso term ${\sum }_{k = 1}^{m} || P_{H}^{(k)} ||_{2}$ is the sum of the *ℓ*_2_-norm per column which is further minimized for *α* > 0 if each individual *ℓ*_2_-norm is kept as small as possible. Indeed, this means that at each time/time-scale snapshot, spatial sparsity is still promoted.^2^ Standard packages exist for multitask elastic-net problems, of which the implementation in glmnet [31] was used. The parameters *α* and *λ* in (6) were determined by an exhaustive parameter search as explained in Section 2.5 and illustrated in Fig. 4.

From the resulting wavelet coefficient matrix *P*_*H*_, the desired heart-surface potentials are finally obtained by Φ_*H*_ = *P*_*H*_*W*^− 1^, where for the inverse wavelet transform it holds that *W*^− 1^ = *W*^*T*^ in the case of the orthogonal wavelet transform, which boils down to using the time-reverse filters as reconstruction filters. The approach is visualized in Fig. 2.

### In vivo recordings and non-invasive reconstruction

**Fig. 3:**
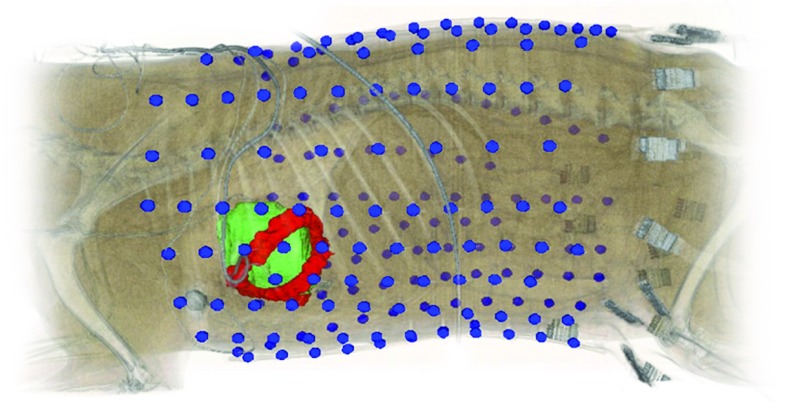
Setup of the canine experiments. Body-surface potentials were recorded with 192 electrodes (blue) and epicardial potentials with 99 implanted electrodes (red). A CT scan was performed to localize the electrodes and epicardial surface (green)

In vivo data were acquired in a canine experiment, illustrated in Fig. 3. Details on the experimental setup can be found in [5] and are summarized below.

In three normal, anesthetized dogs, 99 electrodes were implanted around the epicardium via a thoracotomy and 192 body-surface electrodes were attached to the torso after chest closure. Potential recordings were obtained simultaneously on the body surface and on the epicardium. Reference electrodes for both recording systems were attached to the lower abdomen. A CT scan was performed and used to digitize a homogeneous geometry which consisted of the body-surface electrodes and the epicardial surface [35]. The transfer matrix, relating the electrical activity at the cardiac surface to the body surface, was computed with methods available from the SCIrun software repository [1]; details of our inverse reconstruction approach can be found in [5]. Beats were recorded during normal sinus rhythm and during epicardial pacing (and limited endocardial pacing).

Epicardial potentials were reconstructed with the wavelet-based multitask elastic-net regularization method described in the previous section. For this, we used the Daubechies-2 (i.e., filter length 4) wavelet. Choosing more vanishing moments seems to have a limited effect on sparsity [19], but improves the bandpass filters which is beneficial for denoising [43]. Some additional details can be found in the Online Supplement.

We used three levels of wavelet decomposition, which provided results that were as accurate as when more levels were used, but computationally more efficient. We chose the stationary-wavelet approach over the discrete-wavelet approach because of its time-invariant properties. Specifically, the implementation that is available in Matlab [23] was employed. The optimization of (6) was performed with the glmnet package in Matlab [23, 31].

### Post-processing

From non-invasively reconstructed electrograms, activation and recovery times were determined by considering the maximum negative slope (max−*∂*Φ_*H*_(*t*)/*∂t*) during activation and the maximum positive slope (max*∂*Φ_*H*_(*t*)/*∂t*) during recovery. To reduce the influence of noise, we fitted a smoothing spline curve to each electrogram before differentiating. Activation and recovery times obtained with our approach could then be compared to the invasively obtained timings of activation and recovery.

The origin of an epicardially paced beat was defined as the epicardial node with the earliest reconstructed activation time. This location was then compared to the known location of pacing.

### Analysis of performance

**Fig. 4:**
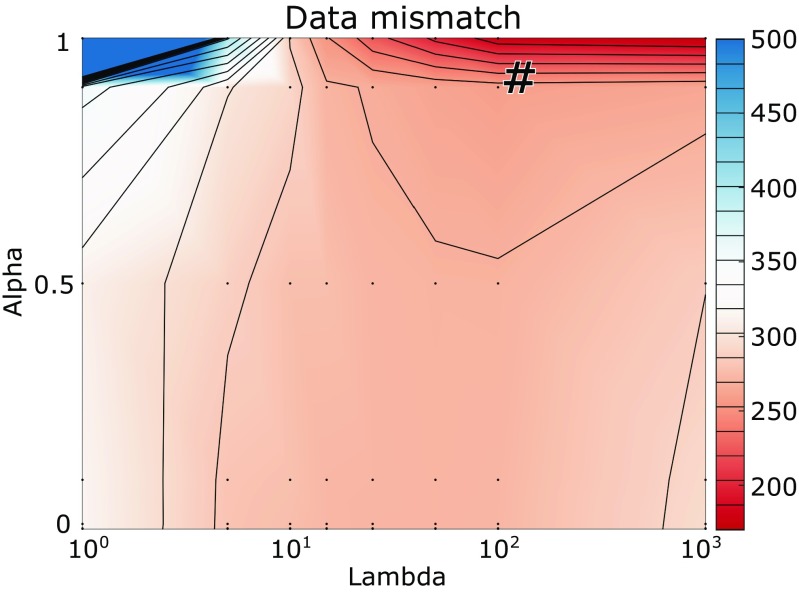
Dependency of in vivo results on elastic-net algorithm parameters, based on 8 recorded beats in a dog. For each combination of alpha and lambda, the data mismatch is shown in microvolts. Grid search points are indicated by black dots. The number sign denotes the value ultimately chosen for the remainder of the analyses (*α* = 0.9,*λ* = 100)

Given the multitask elastic-net model in (6), a grid search was carried out to identify an optimal point in the two-dimensional parameter space given by the regularization parameter *λ* and the elastic-net mixing parameter *α* (see Fig. 4). Optimality was defined in terms of data mismatch, i.e., the mismatch between the recorded body-surface potentials and body-surface potentials provided by the forward solution based on reconstructed epicardial potentials as in (1). The data mismatch in inverse problems is usually a suboptimal criterion and more advanced approaches (such as the L-curve method [15]) are employed to select the optimal parameter, but are not designed for two-parameter problems as the one in our approach. For this paper, we decided to focus on the novelty of the method itself and leave its parameter selection for future study.

Due to the computational cost, the grid search was determined in a subset of 8 beats only.

After identifying optimal values for *λ* and *α*, we used the corresponding model to assess performance of the proposed approach. Performance was assessed in terms of accuracy of:

Reconstructed epicardial potentials.: For each epicardial electrode, Pearson’s correlation coefficient (CC) was computed between the recorded electrogram and the reconstructed electrogram at the corresponding (closest) virtual epicardial node.
Estimated activation and recovery time.: Linear correlation between recorded and reconstructed activation/recovery timings was assessed by means of Pearson’s correlation coefficient.
Estimated locations of pacing.: Localization error (LE) was defined as the Euclidean distance between the reconstructed location of earliest activation and the known pacing location.

Results were statistically compared with Wilcoxon signed-rank tests (for paired measurements) or Wilcoxon rank-sum tests (for unpaired measurements). Performance of the proposed approach was compared with Tikhonov zeroth-order regularization, one of the most commonly used regularization approaches in the inverse problem of electrocardiography [5, 7].

## Results

In the grid search with 8 beats, the point of lowest mismatch was for *α* = 1. At this point, there is no effect of the Tikhonov term of Eq. 6. Additionally, we noticed that the correlation coefficients with the recorded signals improved with slightly lower values of *α* (see Online Figure 5). Therefore, we chose a point close to but not at the minimum data mismatch: *α* = 0.9 and *λ* = 100 (indicated by the number sign in Fig. 4). These parameter settings were then used for the full in vivo analyses.

**Fig. 5:**
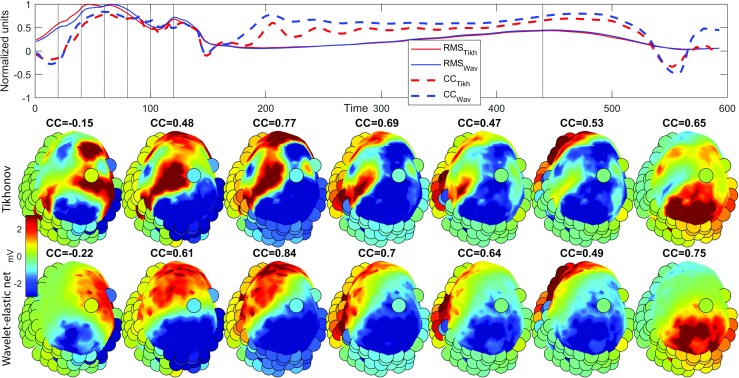
Reconstructed potentials on the epicardial surface at specific time points during a single (paced) beat. Top row: The root mean square (RMS) of the Tikhonov-reconstructed epicardial potentials (red) and the RMS of the Wavelet-elastic-net-reconstructed potentials (blue). The first peak is the QRS complex, while the second wave is the T wave. The spatial CC (per time instant) between all reconstructed potentials and the invasively recorded potentials is depicted as well for both methods (dashed lines), indicating an overall higher CC for the proposed method. The second and third row show potentials at specific time instants (moments are visually indicated by dashed lines in the top row). The colored heart surface displays the *reconstructed* potentials (second row: Tikhonov; third row: wavelet-elastic net), while the colored circles display the *recorded* potentials at the invasive electrodes

Figure 5 shows an example of a few potential distributions of the recorded and reconstructed potentials with the Tikhonov method and wavelet-domain multitask elastic-net method. The latter method obtains overall higher CC and a more realistic potential pattern (i.e., it shows a less patchy pattern, which is to be expected for a paced beat).

**Fig. 6:**
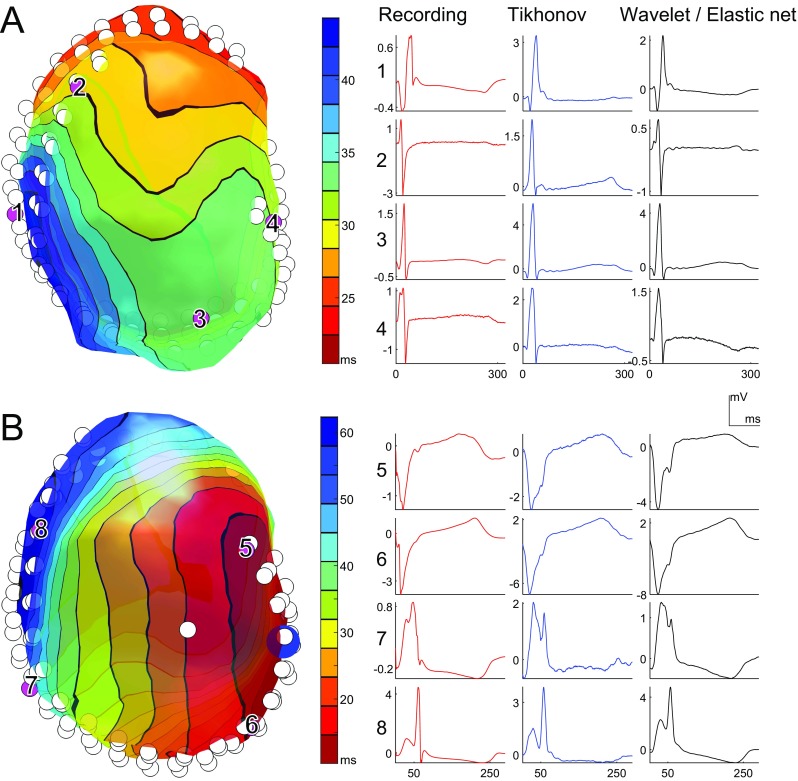
Apical view of the ventricular epicardium (left, colored according to non-invasively reconstructed activation times) and recorded and reconstructed electrograms (right) during a sinus beat (panel A) and a left-ventricular paced beat (panel B, pacing location indicated by blue sphere). White circles represent the implanted epicardial electrodes. For selected electrodes (purple, numbered), the corresponding electrograms are shown: recorded (red), Tikhonov reconstructed (blue), and wavelet-domain multitask elastic-net reconstructed (black).

Panel A of Fig. 6 shows an example of reconstructed activation times on the ventricular epicardium for a sinus beat. For selected electrodes, the corresponding electrograms are shown as recorded, Tikhonov reconstructed, and wavelet-domain multitask elastic-net reconstructed. Panel B of Fig. 6 shows similar results for a left-ventricular paced beat. In general, wavelet-domain multitask elastic-net regularization was able to recover some details (e.g., the initial positive deflection in the QRS complex of electrode 1, and the terminal negative deflection in electrode 2) that were lost with Tikhonov regularization.

**Fig. 7:**
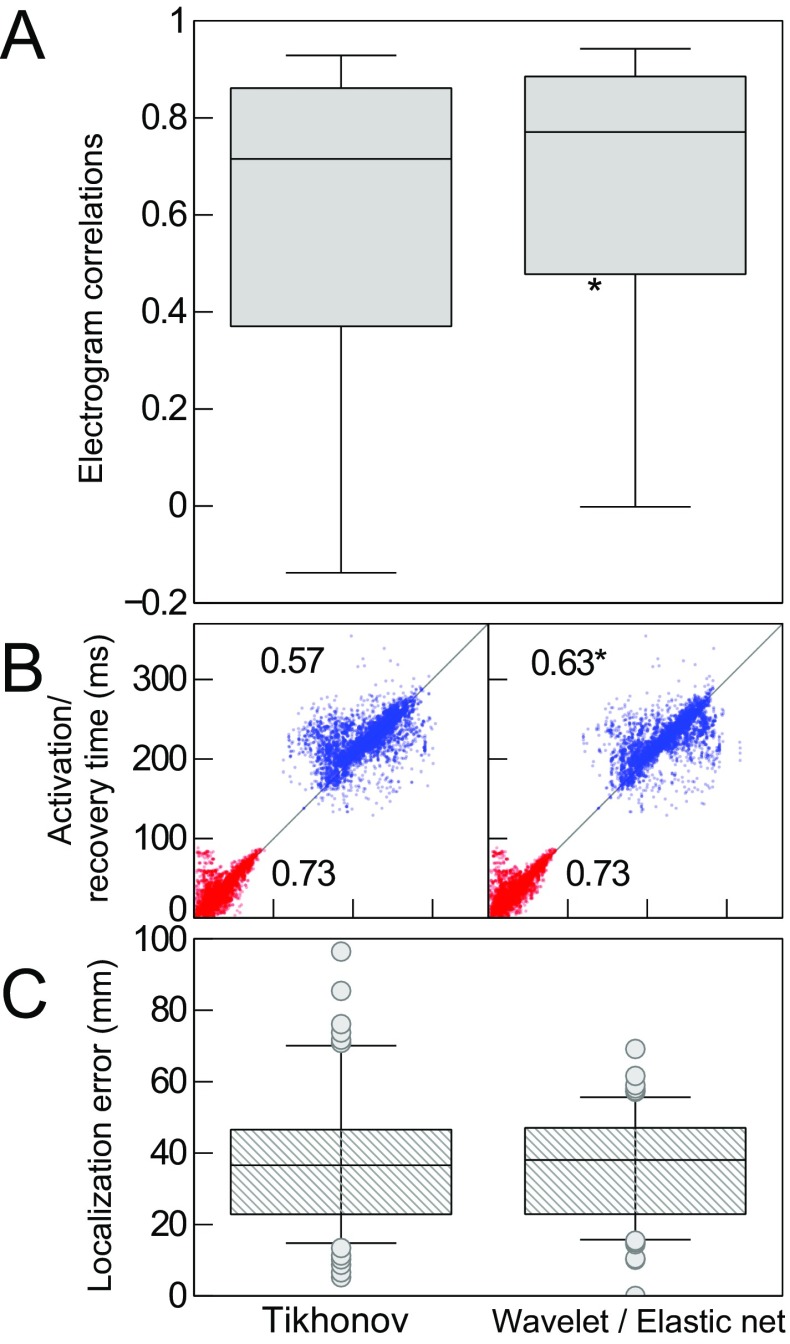
Results for the full data set. Columns show the results for the different reconstruction methods: traditional Tikhonov regularization, or wavelet-domain multitask elastic-net regularization. Panel A: box plots of correlation coefficients between recorded and reconstructed electrograms. Box spans the interquartile range (IQR), i.e., the 25–75% range; median indicated by horizontal line; whiskers at 9–91% range. Wavelet-based multitask elastic-net regularization improves reconstruction quality. Panel B: Activation times (red) and recovery times (blue) as reconstructed (horizontal axes) vs. recorded (vertical axes). Recovery times, especially, are improved by wavelet-based multitask elastic-net regularization. Panel C: Localization error between detected and known origins of pacing. An asterisk indicates significant improvement compared to Tikhonov results

In the in vivo experiments, 89 beats were recorded, with on average 60 epicardial electrodes per beat recording high-quality electrograms as ground truth. Figure 7 shows results for this full data set. Columns show the results for the different reconstruction methods: Tikhonov regularization or wavelet-based multitask elastic-net regularization. Panel A shows accuracy of reconstructed epicardial potentials in terms of correlation coefficients between recorded and reconstructed electrograms. Wavelet-based multitask elastic-net regularization is able to recover more details in the electrograms, significantly improving reconstruction quality (CC_Tikh_ = 0.72,CC_W/E_ = 0.77,*p* < 0.05), although distributions overlap considerably. Panel B shows activation times and recovery times as reconstructed vs. recorded. Activation times (red) are not improved by wavelet-based multitask elastic-net regularization, but recovery times are improved significantly (R_Tikh_ = 0.57,R_W/E_ = 0.63,*p* < 0.05). Panel C shows localization error between detected and known origins of pacing. There is no significant difference between the two regularization methods, although it appears that there are fewer outliers with the new method.

## Discussion

**Fig. 8:**
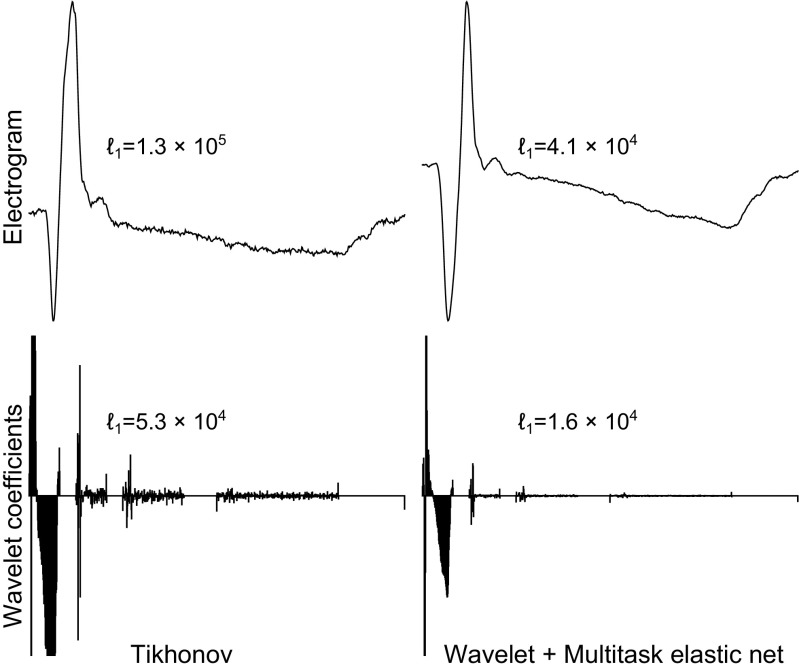
Left column: plots and *ℓ*_1_-norms of an epicardial electrogram reconstructed with Tikhonov regularization (top left) and its wavelet coefficients (bottom left; from approximation coefficients on the left, to finer and finer detailed coefficients towards the right). Right column: plots and *ℓ*_1_-norms of electrogram reconstructed with wavelet-domain elastic net (top right) and its wavelet coefficients (bottom right). Note the higher sparsity (lower *ℓ*_1_-norm) for the novel approach

We have introduced a new method to regularize the inverse problem of electrocardiography by pursuing sparsity of its wavelet representation in both time and space. Figure 8 illustrates that, in terms of *temporal sparsity*, a representation of the signal in the wavelet domain is more efficient than a representation in the time domain. A direct comparison with wavelet decompositions of the recorded electrograms is problematic since the epicardial electrograms are recorded with a different recording system. Additionally, sparsity is further promoted by our proposed multitask elastic-net approach. Online Figure 1 similarly illustrates that *spatial* sparsity is higher in the wavelet domain. An example of the wavelet decomposition of a beat and the sparsity of the coefficients on different scales is shown in Online Figure 2. It is important, however, to realize that the goal is to obtain an accurate inverse reconstruction (for which we proposed a sparsity-based approach in a representative domain), not to obtain a sparse reconstruction per se.

Epicardial potentials reconstructed with this wavelet-domain multitask elastic-net approach attain a higher correlation coefficient compared to traditional zeroth-order Tikhonov regularization. More importantly, estimation of recovery time was also improved by the proposed method. Abnormalities in recovery times can be an important substrate for cardiac arrhythmias and sudden cardiac death [14]. Improved recovery time imaging might thus improve clinical care, but further research on the relevance of this finding in diseased states (such as long QT syndrome, sudden cardiac death, etc.) is needed. Currently, simultaneous invasive and body-surface recordings in such diseases are not available.

Activation time imaging is not improved and consequently localization of beat origin (which was based on the earliest activation time) is not improved either, although the novel method seems to result in fewer outliers.

Our results about the optimal value for alpha (alpha = 0.9) show that (6) weights the 1-norm term 18 times more heavily than it does the Tikhonov term, suggesting that the multitask Lasso component is most beneficial for the dataset at hand. However, the optimal value of alpha may still be strongly dependent on the specific dataset, and the suggested formulation in (6) provides a more general setting which can better adapt to different datasets.

The multitask elastic net returns the inverse solution which has a sparse group structure over the wavelet coefficients. This means that optimization is performed over both time-frequency (columns of *P*_*H*_, wavelet coefficient index) and space (rows of *P*_*H*_, the epicardial nodes, by group structure). The spatial relation between the epicardial nodes is implicitly included in this process, as this relation is captured by the transfer matrix *A*. However, this relationship could be exploited even more if one could define a wavelet transform over the irregularly curved heart surface.

To the best of our knowledge, spatiotemporal sparsity in the wavelet domain has not been pursued previously in a regularization method for ECGI. Previous studies have proposed alternatives to promote spatial or temporal (or both) sparsity of the inverse solution (outside the wavelet domain) [8, 11, 24, 41]. All those methods require prior assumptions based on the properties of the electrophysiology of wavefront propagations, which are exploited to regularize the inverse solution. Since the method we propose aims to promote spatiotemporal sparsity of the inverse solution, those properties are still accounted for when looking for a sparse solution, given the spatiotemporal nature of cardiac propagation and recovery. At the same, since those properties are not directly introduced as constraints in our approach, we speculate that our method may be more data driven and versatile. Another study investigated the possibility of using *ℓ*_*p*_-norm regularization to bridge the gap between the scattered solution of *ℓ*_1_ regularization, useful to detect sparse and focal sources of activation and pacing sites, and the smeared solution of *ℓ*_2_ regularization, which provides a better approximation of extended source regions [32]. In this respect, a question that arises is whether our method could be generalized to use *ℓ*_*p*_ regularization, and it will be investigated in a future study. Greensite SVD applies spatiotemporal regularization (without pursuing sparsity) [12], but was outperformed by zeroth-order Tikhonov regularization in a previous study by our group [4] (although other studies find different results). Temporal sparsity was pursued in a different formulation of the inverse problem of electrocardiography [42] but lacked a spatial component, and focused only on activation times, not electrogram morphology or recovery times. Additionally, that method was only validated in small animals, although it was able to obtain results throughout the full myocardium, not only the epicardium.

Moreover, wavelets have not been applied in regularization of the inverse problem of electrocardiography. One advantage of wavelet-based regularization is the freedom in choosing the wavelet basis. Here, we chose to apply the Daubechies-2 wavelet transform, which was previously shown to be a good choice for sparse representation of heart-surface potentials amongst orthogonal wavelet transforms (see [19] and Online Figure 3). However, improved results could be expected for different choices for the wavelet basis. We limited our approach to orthogonal wavelets to ensure that a Parseval’s relation exists in (4), i.e., ensuring equivalence between the *ℓ*_2_ fit criterion in the wavelet domain and in the time domain. Within this setting, designed orthogonal wavelets could be used to tailor this method to specific situations [17, 20]. This creates more freedom than using a fixed (semi-)physiological model that cannot be adopted to pathological situations, as in [42]. For example, if one is interested in fractionated electrograms (which could occur after myocardial infarction), our method would allow employing a more fractionated wavelet to possibly better enhance regions of fractionation. Additionally, abnormally long or short activation or recovery durations might be captured with specifically designed wavelets. We were not able to test this in vivo, as the dogs from this experiment had healthy hearts. Also still within the orthogonal framework, multiwavelets [28] can be applied to distinguish between certain morphologies and even specifically designed multiwavelets can be employed [18]. For example, if one would like to include biorthogonal wavelets, the Parseval’s relation is no longer in place and the problem is the time and wavelet domain can differ. Possibly, a normalization might partly remedy this, but one has to be careful that energy is not transferred in an undesirable way.

Future challenges to improve this method include the following: (1) investigation of a more robust way to select the optimal parameters *α* and *λ* than a grid search over the data mismatch; and investigating the effect of determining these parameters per beat, instead of over all beats; (2) investigation of an even wider range of activation and recovery patterns, e.g., septal sources of activation, bundle branch block, increased local dispersion of recovery, the effect of scar; (3) investigation of specifically designed orthogonal wavelets for specific disease types.

## Conclusion

We have introduced a novel method to regularize the inverse problem of electrocardiography. By simultaneously pursuing a sparse wavelet representation in time-frequency and exploiting correlations in space, epicardial potentials were non-invasively reconstructed with higher accuracy than with Tikhonov zeroth-order regularization. This indicates that sparse representations of the cardiac source can help to improve reconstruction accuracy in electrocardiographic imaging. Most notably, our approach led to improved estimation of recovery times, which is important to assess substrate for cardiac arrhythmias. More importantly, this novel technique opens potentially powerful opportunities for clinical application by allowing to choose wavelet bases that are optimized for specific clinical questions.

## Electronic supplementary material

Below is the link to the electronic supplementary material.

(PDF 634 KB)

## Footnotes

1 This is convenient from a notation viewpoint. The convolution matrix *W* does not have to be implemented explicitly. It is more convenient to use a filter bank for performance reasons.

2 For an illustration, see the online supplement.
