# Supplementary material for: Wavelet-promoted sparsity for non-invasive reconstruction of electrical activity of the heart
Source: Med Biol Eng Comput. 2018 May 12;56(11):2039–50. doi: 10.1007/s11517-018-1831-2 (PMC6208718; doi:10.1007/s11517-018-1831-2)
Supplement: Supplementary file 1 — (PDF 634 KB) [file 11517_2018_1831_MOESM1_ESM.pdf]

Online Supplement

This online supplement accompanies the paper "Wavelet-promoted sparsity for noninvasive reconstruction of electrical activity of the heart" by M. Cluitmans, J. Karel, P. Bonizzi, P. Volders, R. Westra, and R. Peeters.

Contents

Topic 1: Spatial sparsity 2

Topic 2: Wavelet coefficients 3

Topic 3: Wavelet design and wavelet choice 4

Topic 4: Other regularization methods 5

Topic 5: Parameter search 6

Topic 1: Spatial sparsity

In Online Figure 1, the spatial sparsity is depicted for a single (sinus) beat. The spatial sparsity is the L1-norm of the potential distribution per time instant (when in the potential domain) or over the columns of the wavelet coefficient matrix (when in the wavelet domain). Clearly, the spatial sparsity is much lower in the wavelet domain (and similar between the Tikhonov and Wavelet-Elastic-Net method). This supports our thesis that sparsity in the wavelet domain might be a suitable criterion.

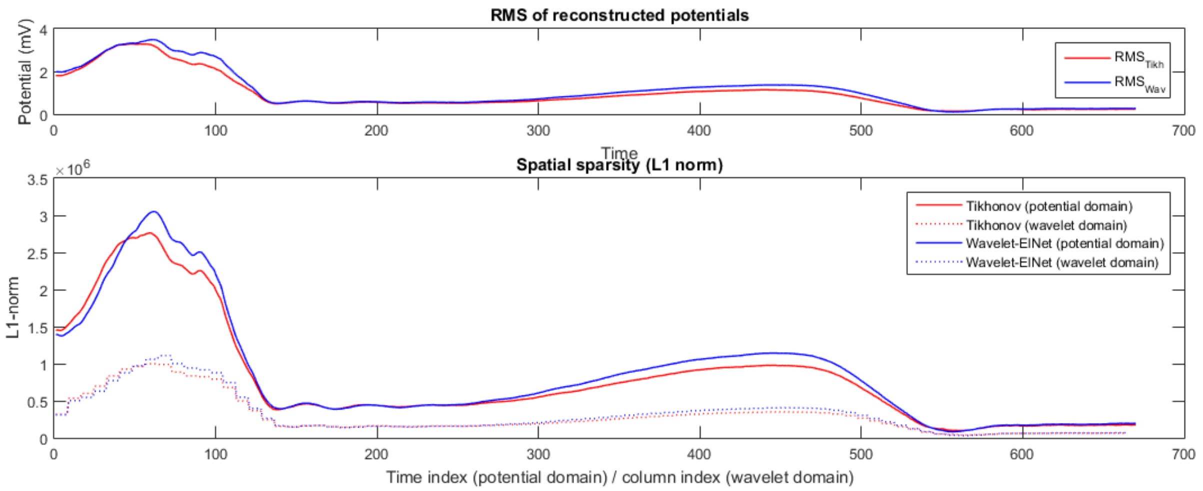

Online Figure 1: Spatial sparsity of reconstructed potentials. Top row: The root mean square (RMS) of all reconstructed potentials with the Tikhonov method (red) and the Wavelet-Elastic-Net method (blue), to visualize the general course of the potential distribution (first peak: QRS complex; second peak: T wave). Bottom row: the L1 norm for the reconstructed potentials (i.e., potential domain: full lines) and their wavelet decomposition (i.e., wavelet domain: dotted lines), for the Tikhonov approach (red) and the Wavelet-Elastic-Net approach (blue).

Topic 2: Wavelet coefficients

Online Figure 2 displays the wavelet coefficients for a single beat, to illustrate the effect of wavelet decomposition at different scales. Note that many coefficients have a low (or zero) value and that this wavelet representation is thus an efficient (sparse) choice.

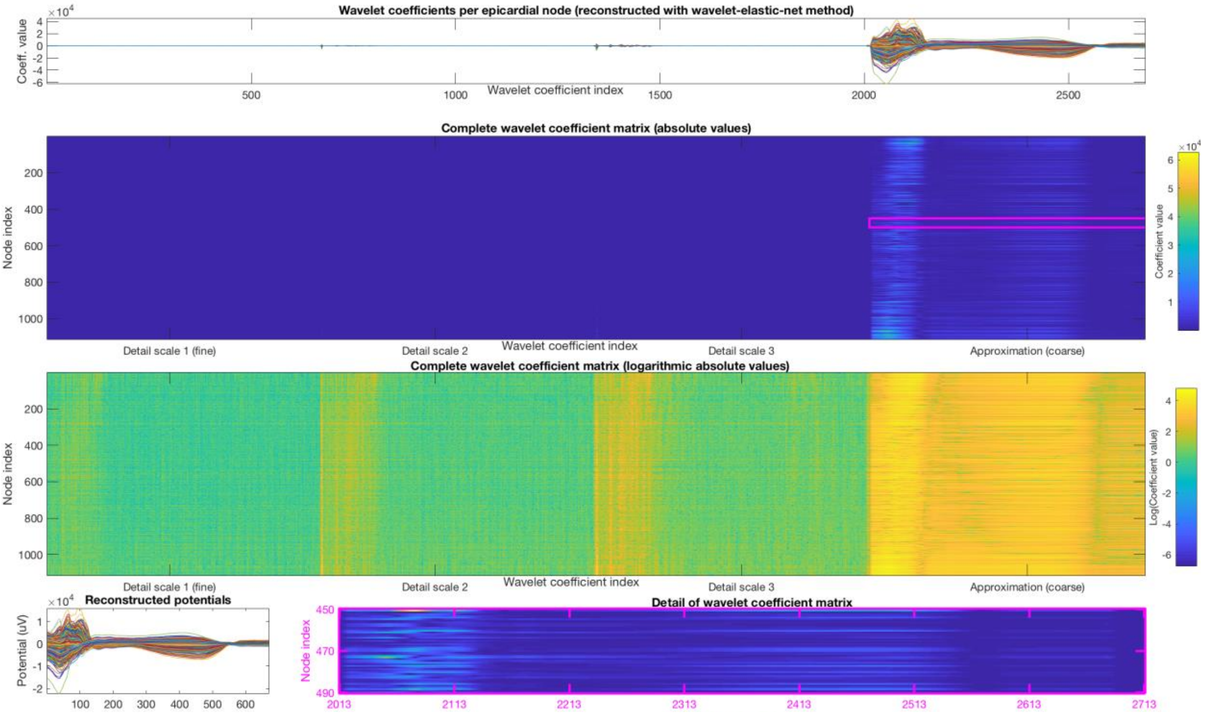

Online Figure 2: Example of the wavelet representation of epicardial potentials.
Top row: the wavelet coefficients (as stacked from detail level 1, to detail level 2, to detail level 3 and approximation coefficients), superimposed for each epicardial node.
Second row: the wavelet coefficients now displayed as matrix, showing the coefficient value in color per epicardial node (rows) and per wavelet column.
Third row: identical data but now with a logarithmic scale of the absolute values, to show that the detail levels contain data as well. Note the many low values (both column-wise and row-wise) in this wavelet representation, indicating sparsity.
Fourth row, left: Reconstructed epicardial potentials. Fourth row, right: zoom-in of the wavelet coefficients, again highlighting the many low values (sparsity).

Topic 3: Wavelet design and wavelet choice

To determine which wavelet was appropriate for this approach, 16 arbitrary beats of canine epicardial electrograms from a healthy control dog in sinus rhythm were concatenated, and the approach from [17, 20] was used to design an 8th order wavelet filter with two vanishing moments enforced, yielding two degrees of freedom. For the wavelet design a stationary wavelet transform was used to ensure shift invariance, which was weighted per scale to ensure that Parseval's relation holds. The design goal was the minimization of the L2 norm of the wavelet representation of the prototype signal. As described in [17,20] a local search was employed using 250 random starting points. The prototype signal, the wavelet representation, its absolute value and the wavelet and scaling function are shown below:

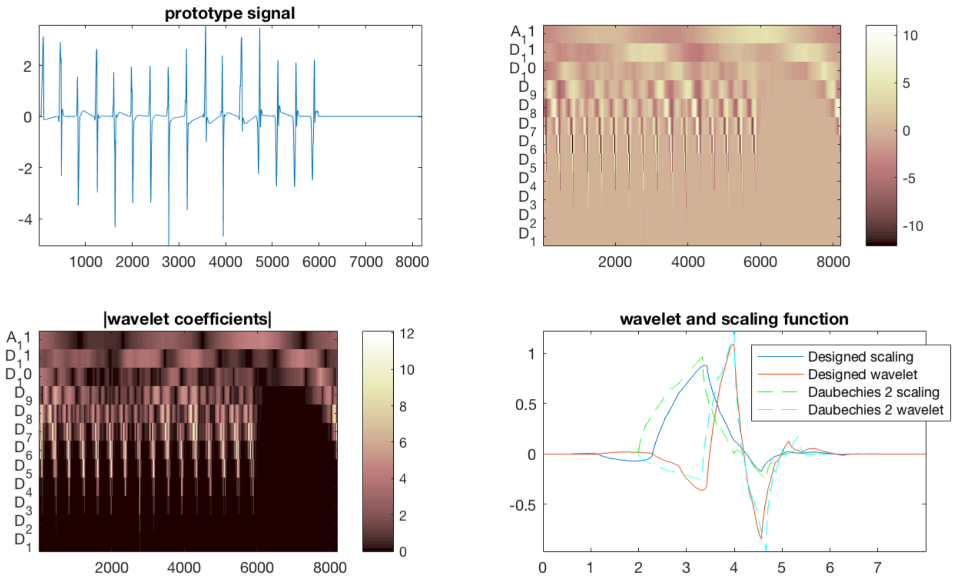

Online Figure 3: The wavelet-design approach, showing the prototype signal (top-left), the wavelet representation (top-right), its absolute value (bottom-left) and the wavelet and scaling function (bottom-right).

Four of the obtained scaling filter coefficients are close to zero and the remaining coefficients are close to the Daubechies 2 scaling coefficients. Therefore, in the main manuscript we worked with the Daubechies 2 wavelet and omitted discussing this wavelet design approach. Nevertheless, in future applications, this approach could be used to design a wavelet for specific cardiac pathologies.

Topic 4: Other regularization methods

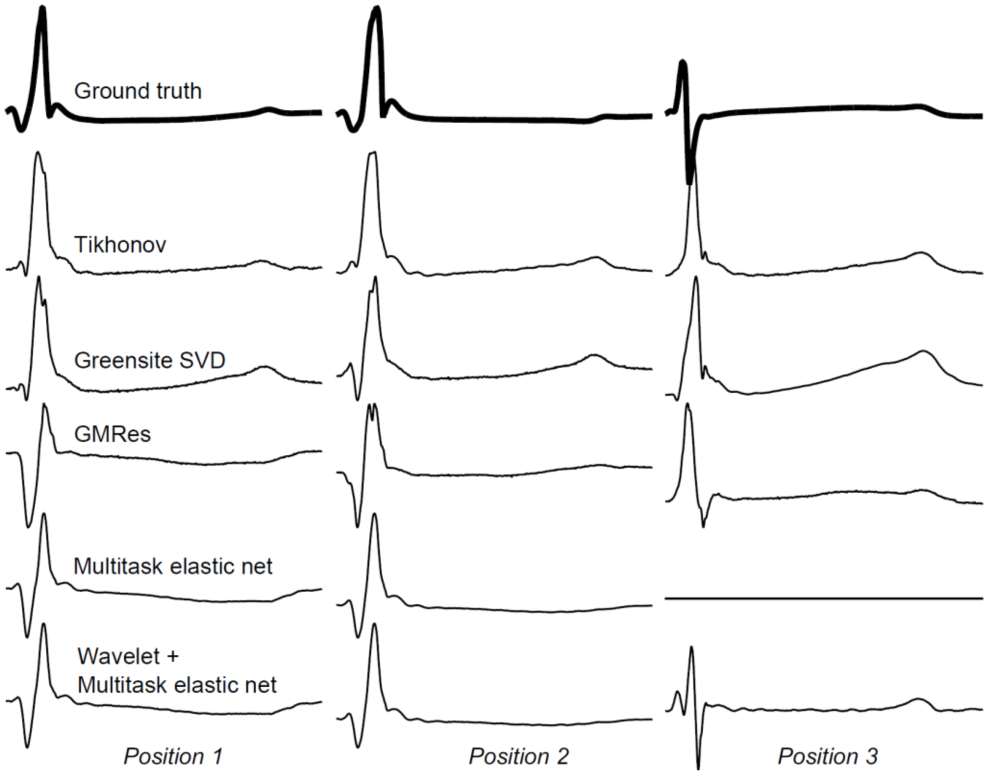

Online Figure 4: Qualitative comparison between different regularization methods.

Tikhonov remains the de facto standard method for inverse reconstruction in ECGI. This makes it the first choice to compare new methods to. In one of our previous studies[1], we compared Tikhonov regularization with Greensite SVD[2] and the Generalized Minimal Residual method (GMRes)[3] and found that Tikhonov showed better performance. For the current study, we performed a qualitative comparison in a single beat of the proposed method (wavelet + multitask elastic net) with multitask elastic net (without wavelet transform), Greensite SVD, and GMRes, and we compared the reconstructed electrograms with the invasive electrograms (ground truth). The comparison is shown in the image above. Note that when the multitask elastic net is applied in the time domain (i.e., without wavelet transform), the sparsity of the signal is enforced partially by reducing some leads to zeros only. This does not occur when enforcing multitask elastic net on the wavelet coefficients.

References for Topic 4

1: Cluitmans, M., Peeters, R., Volders, P. & Westra, R. Realistic Training Data Improve Noninvasive Reconstruction of Heart-Surface Potentials. in Conf Proc IEEE Eng Med Biol Soc. 6373–6376 (IEEE, 2012).
2: Greensite, F. & Huiskamp, G. An improved method for estimating epicardial potentials from the body surface. Biomedical Engineering, IEEE Transactions on 45, 98–104 (1998).
3: Ghosh, S. & Rudy, Y. Accuracy of quadratic versus linear interpolation in noninvasive Electrocardiographic Imaging (ECGI). Ann Biomed Eng 33, 1187–1201 (2005).

Topic 5: Parameter search

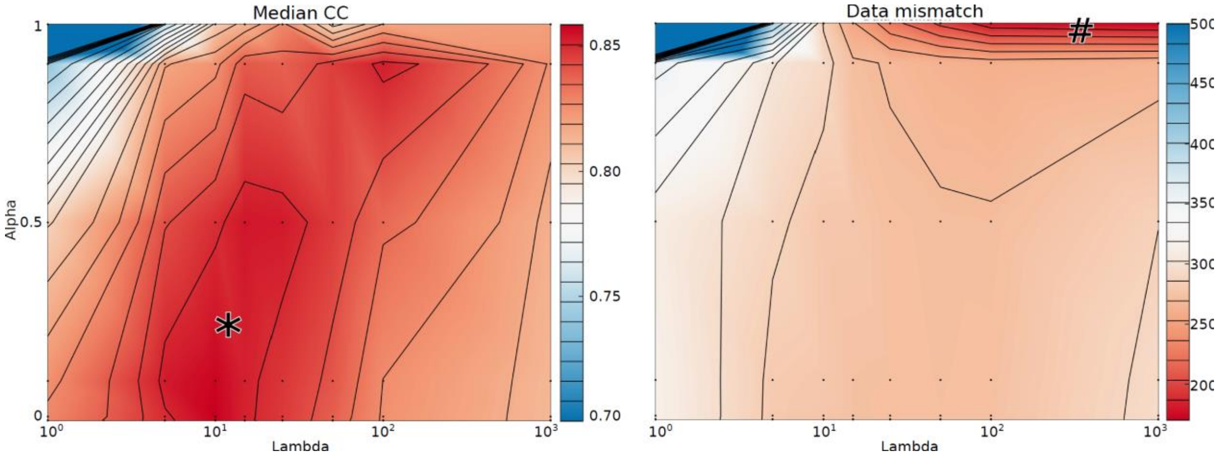

*Online Figure 5: Dependency of in vivo results on wavelet-elastic-net algorithm parameters, based on 8 recorded beats in a dog. For each combination of alpha and lambda, the median correlation coefficient (CC, left) and data mismatch (right) is shown. Red color indicates optimal results, the asterisk * the highest correlation coefficient, and the hash # the lowest data mismatch. Grid search points are indicated by black dots. Since CC can only be computed when ground truth data are available, the data mismatch is a more honest method of selecting parameters.*
